# Occupational silica exposure drives systemic immune dysregulation and tumor microenvironment susceptibility: evidence from a real-world study

**DOI:** 10.3389/fimmu.2026.1775236

**Published:** 2026-03-02

**Authors:** Gao Hao, Zaitian Zhang, Hui Zhang, Qiang Luo, Jingyi Zhao, Liu Baishan

**Affiliations:** 1 School of Public Health, North China University of Science and Technology, Tangshan, China; 2 Department of Orthopedics, First Affiliated Hospital of Hunan University of Medicine, Huaihua, China; 3 Department of Rheumatology and Immunology, Children’s Hospital of Chongqing Medical University, Chongqing, China; 4 Institute of Traditional Chinese Medicine, Chengde Medical University, Chengde, China; 5 Department of Respiratory, Jiulongpo District People’s Hospital of Chongqing, Chongqing, China

**Keywords:** carcinoembryonic antigen (CEA), occupational exposure, silica dust, tumor microenvironment, tumor-associated macrophages

## Abstract

**Background:**

Occupational exposure to carcinogenic dusts such as silica is a well-established risk factor for cancer. However, the molecular mechanisms linking early exposure to tumor-promoting microenvironmental changes remain poorly defined. Emerging evidence suggests that chronic immune dysregulation and remodeling of the tumor microenvironment (TME) may serve as critical intermediates.

**Methods:**

We analyzed occupational health data from 5,482 industrial workers in Anhui Province, China. Explainable machine learning models were constructed using exposure profiles and hematological immune parameters to predict carcinoembryonic antigen (CEA) positivity, with feature contributions interpreted via SHAP values. Experimental validation involved silica-stimulated THP-1 monocytes and colorectal cancer (CRC) cell lines to assess inflammatory activation and paracrine regulation of CEA. Silica- and CRC-associated genes were integrated from public databases to construct protein–protein interaction networks, identify hub genes, and evaluate prognostic significance using TCGA and GSE39582 datasets. Single-cell RNA sequencing (scRNA-seq) analysis was used to resolve cell type–specific expression patterns.

**Results:**

Among 14 algorithms tested, CatBoost exhibited the highest predictive performance for CEA positivity. SHAP analysis highlighted the monocyte-to-lymphocyte ratio and silica exposure as dominant contributors. Mediation analysis confirmed that systemic inflammation partially mediated the silica–CEA association. *In vitro*, silica activated NF-κB–dependent IL-6 secretion in THP-1 cells, and conditioned media dose-dependently upregulated CEA expression in CRC cells—an effect attenuated by NF-κB inhibition or IL-6 neutralization. Multi-omics analysis identified 42 overlapping genes linking silica exposure to CRC, with enrichment in cytokine signaling, adhesion, and matrix remodeling pathways. A hub gene–based risk score was significantly associated with overall survival. scRNA-seq analysis revealed elevated expression of inflammation- and adhesion-related genes in tumor-associated macrophages.

**Conclusions:**

Occupational silica exposure induces macrophage-driven inflammatory signaling that promotes early CEA elevation and TME remodeling. Integrating machine learning with experimental and multi-omics validation provides a translational framework for identifying exposure-responsive biomarkers and immune-related cancer risk in occupational settings.

## Introduction

Occupational carcinogens are an important but historically unrecognized component of the global cancer burden (1). With the increasing specialization and diversification of industrial chemicals, workers are exposed to more and more new complex toxins, which has raised concerns about the long-term health consequences of occupational exposure (2). Epidemiological evidence suggests that approximately 2% -8% of cancers can be attributed to occupational exposure, highlighting its significant impact on public health (1, 3). In 2000, the World Health Organization’s Comparative Risk Assessment (CRA) project provided the first global systematic estimate of the cancer burden caused by occupational exposure (1). On this basis, the International Agency for Research on Cancer (IARC) classifies nearly 200 carcinogens as Group 1 or 2A human carcinogens, many of which are commonly present in work environments (4).

Common occupational carcinogens include asbestos, crystalline silica, sawdust, polycyclic aromatic hydrocarbons, heavy metals, aromatic amines, organic solvents, and environmental pollutants such as formaldehyde and radon (4–6). These drugs are causally associated with a range of malignant tumors, including lung cancer, mesothelioma, bladder cancer, leukemia, and skin cancer (5–7). Importantly, the carcinogenic risk associated with occupational exposure not only reflects the inherent genetic toxicity of individual factors, but also factors such as the duration and intensity of exposure, co exposure, and long incubation periods (6, 8). Occupational exposure occurs in different industries, including rubber and pigment manufacturing, textile production, mining, painting, insulation, and metal processing (5). Due to the fact that many occupational cancers only appear decades after initial exposure, early detection and attribution remain significant challenges (8). It is estimated that approximately 150000 deaths worldwide each year are related to historical occupational exposure to some recognized carcinogens (1, 3). Despite regulatory measures such as bans, exposure restrictions, and monitoring systems, occupational cancer continues to exacerbate the global cancer burden and requires ongoing mechanism investigations and policy attention (5).

Traditional occupational cancer research emphasizes direct genetic toxicity mechanisms, including DNA damage and mutation accumulation caused by carcinogens (6, 9). However, new evidence suggests that non genotoxic mechanisms also play a critical role in tumorigenesis by shaping the local tissue environment (10). Long term exposure to substances such as asbestos, benzo [a] pyrene, polycyclic aromatic hydrocarbons, and certain metals can cause persistent inflammation, oxidative stress, and immune dysfunction, leading to abnormal activation of stromal cells, immune cells, and endothelial cells (9–11). These reactions contribute to the formation of a tumor microenvironment (TME) characterized by immune evasion, angiogenesis, extracellular matrix remodeling, and metabolic reprogramming (10–12). Different exposure types may drive specific TME phenotypes; For example, the accumulation of silica and asbestos in lung tissue is associated with polarization of M2 macrophages and activation of fibroblasts, promoting fibrosis and lung cancer progression (11, 13), while exposure to benzene and aromatic amines is associated with immune failure and epigenetic changes in bone marrow and bladder tissue, promoting an environment favorable for the development of leukemia or urothelial carcinoma (14).

In this context, identifying circulating biomarkers that reflect early exposure effects and TME remodeling has become a priority in occupational cancer research (6, 10). Carcinoembryonic antigen (CEA) is one of the earliest discovered and most widely used tumor markers, showing sensitivity and clinical relevance in various cancers associated with occupational exposure (15, 16). CEA levels are generally low in normal adult tissues, but increase in epithelial malignancies such as lung cancer, gastric cancer, pancreatic cancer and bladder cancer; Its expression is not only related to tumor burden, but also to tumor immune microenvironment and tumor heterogeneity (16, 17).

More and more evidence suggests that elevated CEA may be an early indicator of chronic inflammatory response and transformation processes in occupational exposure populations, and may reflect dynamic changes in TME caused by exposure (7, 17). For example, exposure to asbestos or silica dust is associated with activation and M2 polarization of alveolar macrophages, which may be consistent with increased CEA expression and immunosuppressive environment (13, 17), while benzene and its metabolites are associated with bone marrow suppression and immune failure, which is related to elevated circulating CEA (7, 14). These findings suggest that CEA can not only serve as a static disease monitor, but also as a ‘liquid sensor’ that captures the continuous process from exposure to TME regulation to malignant tumors, enabling early detection and risk stratification at the individual level (7, 17, 18).

In this context, we attempt to elucidate the relationship between occupational exposure and CEA in large worker cohorts. Firstly, we will apply interpretable machine learning to a comprehensive occupational dataset, jointly model heterogeneous exposures, quantify their contributions to CEA changes, and develop cross validated personalized risk prediction models. Secondly, guided by model insights, we conducted *in vitro* validation studies to investigate the biological link between key occupational exposure factors and tumorigenic microenvironment characteristics, such as macrophage activation.

## Methods

The occupational health monitoring data comes from Anhui Province, China, including 5482 workers who participated in routine occupational health monitoring. Individuals with missing key data (CEA, immune parameters, or exposure records) were excluded. In addition, to minimize confounding, participants with a known history of malignant tumors, chronic liver diseases (e.g., cirrhosis, hepatic insufficiency), or documented acute or chronic infectious/inflammatory conditions at the time of examination were excluded based on medical records and clinician review. Workers with abnormal liver enzymes exceeding 3× ULN were also excluded from the analysis. The exposure records in the workplace are systematically linked to standardized hematological test results, enabling a comprehensive assessment of environmental exposure and biological reactions. This study used a cross-sectional design. Each participant contributed a single record, selected from their most recent occupational health examination with complete data on exposure, blood parameters, and tumor markers.

Hematological parameters include platelet count, hematocrit (PCT), mean platelet volume (MPV), red blood cell count, hemoglobin concentration, mean corpuscular hemoglobin (MCH), mean corpuscular hemoglobin concentration (MCHC), and red blood cell distribution width. White blood cell related measurements include absolute counts and corresponding percentages of neutrophils, lymphocytes, monocytes, eosinophils, and basophils. In addition, composite inflammatory indicators were derived, including neutrophil to lymphocyte ratio (NLR), monocyte to lymphocyte ratio (MLR), and systemic immune inflammatory index (SII), to capture systemic inflammatory status.

Occupational exposure variables cover various common hazards in industrial environments. Exposure to particulate matter includes coal dust, crystalline silica dust, dust, other dust, other inorganic dust, welding fumes, cement dust, rock dust, gypsum dust, ceramic dust, limestone dust, marble dust, sawdust, plant dust, and organic dust. Gas exposure includes sulfur dioxide, nitrogen dioxide, carbon monoxide, carbon dioxide, nitrogen oxides, hydrogen sulfide, ammonia, methane, hydrogen cyanide, hydrogen, ozone, chlorine, and hydrogen chloride. Organic solvents and industrial chemicals include benzene, toluene, xylene, naphthalene, phenol, vinyl chloride, dichloroethane, methanol, ethanol, propanol, isopropanol, acetic acid, methyl acetate, ethyl acetate, ethylene glycol, pyridine, ether, dimethyl ether, dimethyl sulfate, and methylamine, as well as hydrazine, hydrazine hydrate, formaldehyde, acetylene, propane, and phosphine. Corrosive and reactive substances include sulfur trioxide, sulfuric acid, hydrochloric acid, nitric acid, sodium hydroxide, potassium hydroxide, sodium carbonate, sodium hypochlorite, polyacrylamide, hydrogen peroxide, disodium hydrogen phosphate, and ammonium sulfate. Heavy metals and highly toxic compounds include mercury iodide, trinitrophenol (picric acid), lead thiocyanate, sodium nitrite, and potassium antimony tartrate. It also recorded complex industrial mixtures, including coke oven emissions and coal tar. Physical occupational hazards include noise, high and low temperatures, arm vibration, ultraviolet radiation, and power frequency electric fields. Acid exposure, alkali exposure, and acid mist or anhydride exposure are recorded as independent occupational categories. Occupational exposure classification was based on a combination of workplace environmental monitoring data and individual job title or position records, as maintained by enterprise occupational health archives. Airborne concentrations of respirable dust and relevant toxicants (e.g., silica, benzene) were assessed through routine annual workplace sampling, and each worker’s exposure status was determined using a 3-year rolling average where available. Exposure levels were evaluated against the national occupational exposure limits (OELs) defined in GBZ/T 229.1–2010 (China). In cases where enterprises implemented stricter internal thresholds, these enterprise-specific limits were adopted accordingly. Workers were categorized as ‘exceeding OEL’ if their exposure exceeded the applicable threshold based on their job function and monitored concentration.

All hematological parameters were measured using standardized protocols under routine occupational health examinations conducted by enterprise-affiliated medical centers. Certified clinical laboratories used automated hematology analyzers with internal and external quality controls. Environmental exposure monitoring followed the national occupational hygiene standard GBZ/T 192.1–2007.

According to the official occupational health records of the company, all occupational exposure variables are encoded as binary indicators (yes/no). Only when long-term workplace monitoring confirms that the exposure level exceeds the corresponding national occupational exposure limit during the monitoring period, will workers be classified as contacts. This binary classification reflects a regulatory based exposure assessment framework that does not capture quantitative exposure intensity.

In addition to the occupational exposure variables and hematological parameters mentioned above, CEA is also listed as an exposure related biomarker. The serum CEA level is obtained from routine occupational health examinations and converted into a binary variable based on the clinical reference threshold of the enterprise. Individuals with CEA values exceeding the upper limit of the normal reference range are classified as CEA positive, while individuals within the reference range are classified as CEA negative. Consistent with the coding strategy used for occupational exposure, CEA is considered a binary variable in all subsequent analyses. Adopting this binary transformation to enhance robustness, reduce the influence of extreme values, and maintain consistency with the classification structure of occupational health monitoring data. CEA positivity was defined using a threshold of 5.0 ng/mL, consistent with the reference range applied by the enterprise clinical laboratory at the time of routine occupational health screening. This threshold aligns with commonly used cut-offs in clinical settings and laboratory assay guidelines. Due to data constraints, the same threshold was applied for all participants regardless of gender or smoking status. While some studies suggest adjusted ranges for specific populations, we treated CEA as a binary outcome using a unified standard and acknowledge this simplification as a study limitation.

This study has been reviewed and approved by the Ethics Committee of North China Institute of Science and Technology (Approval No.: 2024280).

### Explainable machine learning model

We utilized occupational health monitoring data from 5482 workers in a large industrial enterprise in Anhui Province, China, to construct a comprehensive analysis framework for studying the relationship between complex occupational exposure and CEA status. All variables recorded in the occupational health monitoring system are included as candidate features, thereby minimizing prior selection bias to the greatest extent possible. These variables encompass hematological parameters, composite inflammatory indices, and a wide range of occupational exposure indicators, enabling multi-level characterization of host physiology and environmental burden (19).

Hematological measurements capture the global hematopoietic and immune status, including platelet, red blood cell, and white blood cell indices, as well as the relative composition of major white blood cell subgroups. To further summarize systemic immune activation, derived inflammatory markers (NLR, MLR, and SII) were integrated to reflect chronic low-grade inflammation and immune imbalance.

Occupational exposure data covers a highly heterogeneous spectrum of hazards typical of industrial environments. Exposure to particulate matter includes coal dust, crystalline silica, and various inorganic and organic dust, reflecting long-term inhalation risks associated with mining, building materials, and manufacturing processes. Exposure to gaseous and inorganic chemicals includes sulfur and nitrogen oxides, carbon monoxide and carbon dioxide, hydrogen sulfide, ammonia, methane, ozone, chlorine, and related compounds, representing combustion related emissions and confined space exposure. Organic solvents and industrial chemicals encompass aromatic hydrocarbons, halogenated compounds, alcohols, ethers, esters, and other reactive intermediates commonly found in chemical production and material processing. In addition, the dataset also records occupational exposure to corrosive substances, highly toxic substances, and complex industrial mixtures such as coke oven emissions and coal tar. In addition to chemical and particulate hazards, occupational health monitoring systems also systematically record physical stress factors, including noise, extreme heat, arm vibration, ultraviolet radiation, and power frequency electric fields, which constitute sustained non chemical stress factors that can disrupt physiological homeostasis.

A total of 14 algorithms were implemented, covering different method families, including tree based ensemble models (random forest, gradient enhancement, adaptive enhancement, LightGBM, CatBoost), linear and regression based methods (logistic regression, LASSO, partial least squares), kernel and distance based methods (support vector machine with kernel function, k-nearest neighbor), probability and discriminant models (naive Bayes, discriminant analysis), and neural networks (multilayer perceptron). The dataset is randomly divided into a training set (70%) and a testing set (30%). Optimize hyperparameters through 5-fold cross validation using Bayesian optimization (Hyperopt). Evaluate model performance through sensitivity, specificity, and area under the operating characteristic curve (AUC) of the subjects (20, 21). To evaluate robustness, 1000 guided resampling iterations were conducted to obtain the distribution of sensitivity and AUC, and to compare model performance.

Prior to model training, we addressed several key data processing considerations to ensure robustness and fairness. First, given the moderate imbalance between CEA-positive and CEA-negative cases, we applied class weighting schemes for algorithms that support weighted loss functions (e.g., CatBoost and logistic regression), and used stratified sampling throughout training and evaluation steps to maintain representative class distributions.

Second, we evaluated missingness across all features. Variables with less than 5% missing values were imputed using the mean for continuous variables and the mode for categorical variables, while features with greater than 20% missingness were excluded from modeling.

Lastly, during the 1000 iterations of guided resampling, we implemented stratified resampling to preserve the original CEA class proportions across both training and test subsets. This approach minimized bias introduced by artificial class distributions and supported reliable performance comparison across models.

To enhance interpretability, we applied SHAP to quantify the marginal contribution of each feature and characterize potential non-linear exposure–response relationships. Model interpretability was visualized through SHAP summary plots (including beeswarm and bar plots to display global feature importance and direction of effects) and dependence plots (to illustrate non-linear patterns and potential feature interactions).

### Cell lines and culture conditions

Human monocytic THP-1 cells were cultured in RPMI-1640 medium (Gibco, Thermo Fisher Scientific) supplemented with 10% fetal bovine serum (FBS; Gibco) and 1% penicillin–streptomycin (100 U/mL penicillin and 100 μg/mL streptomycin; Gibco). Human colorectal cancer (CRC) cell lines HT29 and Caco-2 were maintained in high-glucose DMEM (Gibco) containing 10% FBS and 1% penicillin–streptomycin. All cells were incubated at 37 °C in a humidified atmosphere with 5% CO_2_. Specifically, the complete culture media composition was described for each cell line, including base medium (RPMI-1640 or high-glucose DMEM), 10% fetal bovine serum (FBS), and 1% penicillin–streptomycin. THP-1 cells were seeded at 5 × 10^5^ cells per well in 24-well plates, while HT29 and Caco-2 cells were seeded at 1.5 × 10^5^ cells per well. After a 12-hour equilibration period, cells were exposed to SiO_2_ for indicated durations: 6 hours for RNA extraction, and 24 hours for cytokine detection. For NF-κB inhibition, cells were pretreated with BAY 11-7082 (5 μM) for 1 hour prior to exposure, and the inhibitor was maintained throughout the stimulation period. Conditioned media (CM) were collected by sequential centrifugation (300 × g, then 2,000 × g), filtered through 0.22 μm membranes, and stored at −80 °C. For stimulation assays, CRC cells were treated with a 1:1 mixture of CM and fresh complete medium to ensure consistent serum and nutrient conditions. In this study, we also used 20–50 nm amorphous SiO_2_ nanoparticles as model particles to induce inflammatory responses; future studies will include respirable crystalline silica to better simulate occupational exposure conditions.

### SiO_2_ particle preparation and dispersion

Amorphous silica (SiO_2_) nanopowder (Sigma-Aldrich, 637238; nominal particle size 20–50 nm) was suspended in sterile phosphate-buffered saline (PBS) to prepare a 10 mg/mL stock solution. Immediately prior to each exposure, the suspension was sonicated in an ice-water bath for 10 min to minimize particle agglomeration and ensure homogeneous dispersion.

### NF-κB inhibition and IL-6 neutralization

NF-κB signaling was inhibited using BAY 11-7082 (Sigma-Aldrich, B5556). BAY 11–7082 was dissolved in DMSO to generate a 10 mM stock solution and applied at a final concentration of 5 μM. Cells were pretreated with BAY 11–7082 for 1 h before SiO_2_ exposure, and the inhibitor was maintained throughout the exposure period.

For IL-6 neutralization experiments, a monoclonal anti-human IL-6 antibody (R&D Systems, MAB206) was added at a final concentration of 2 μg/mL at the onset of stimulation.

### SiO_2_-induced inflammatory activation of THP-1 monocytes

THP-1 cells were seeded in 24-well plates at a density of 5 × 10^5^ cells per well in 500 μL complete RPMI-1640 medium and allowed to equilibrate for 12 h. Cells were then exposed to vehicle or increasing concentrations of SiO_2_ (25, 50, or 100 μg/mL). To evaluate the contribution of NF-κB signaling, cells exposed to the highest SiO_2_ concentration were additionally co-treated with BAY 11-7082, and BAY 11–7082 alone was included as an inhibitor control. The selected SiO_2_ concentrations were determined in pilot experiments to establish a robust inflammatory response while preserving cell viability.

### RNA extraction and quantitative real-time PCR

At 6 h after exposure, THP-1 cells were harvested for RNA isolation. Total RNA was extracted using TRIzol reagent (Invitrogen, Thermo Fisher Scientific) and quantified by NanoDrop spectrophotometry. Complementary DNA was synthesized using the PrimeScript RT reagent kit (Takara, RR037A). Quantitative PCR was performed using PowerUp SYBR Green Master Mix (Applied Biosystems, Thermo Fisher Scientific) on a QuantStudio 5 Real-Time PCR system. Cycling conditions consisted of an initial denaturation at 95 °C for 2 min, followed by 40 cycles of 95 °C for 15 s and 60 °C for 60 s, with subsequent melt-curve analysis. Expression levels of IL6, TNFA, and IL1B were normalized to GAPDH and calculated using the 2^-^ΔΔCt method.

### Cytokine measurement by ELISA

Culture supernatants were collected 24 h after exposure, centrifuged at 2,000 × g for 10 min to remove cellular debris, and stored at −80 °C. IL-6 concentrations were quantified using a human IL-6 ELISA kit (R&D Systems, DY206) according to the manufacturer’s instructions. At this stage, only IL-6 was quantified at the protein level, as it was prioritized for its established role in inflammatory paracrine signaling and tumor-related CEA regulation. Protein-level analysis of IL-1β and TNF-α was not performed, but their mRNA induction was confirmed by RT-qPCR. Future experiments will expand protein validation to include additional cytokines.

### Western blot analysis

Proteins were extracted using RIPA buffer (Cat:R0010, Solarbio) on ice for 30 minutes, then centrifuged at 12,000 rpm for 15 minutes at 4 °C. The supernatant’s protein concentration was measured with a BCA assay (Cat:PC0020, Solarbio). Equal protein amounts (20 μg per lane) were mixed with 5× SDS buffer (Cat:LT101S, Epizyme Biotech), denatured at 100 °C for 10 minutes, and separated by SDS-PAGE using a 5% stacking gel and an 12% separating gel (Cat:P1200, Solarbio), depending on target protein size, at 80 V for 30 minutes and 120 V for 60–90 minutes.

Proteins were transferred to methanol-activated PVDF membranes via wet transfer at 100 V for 60 minutes on ice. Membranes were blocked with 5% non-fat milk in TBST for 1.5 hours at room temperature, then incubated with primary antibodies (Anti-NF-κB p65 Antibody, Cat:A00284-1, Boster) overnight at 4 °C. After three 5-minute TBST washes, membranes were treated with HRP-conjugated secondary antibodies for 1 hour at room temperature. Following another three TBST washes, protein bands were visualized using ECL and quantified with ImageJ, using GAPDH (Anti-GAPDH Antibody, Cat:A00227-1, Boster) as a reference.

### Direct exposure of CRC cells to SiO_2_

HT29 and Caco-2 cells were seeded in 24-well plates at 1.5 × 10^5^ cells per well and allowed to adhere overnight. Cells were subsequently treated with vehicle or SiO_2_ at low, medium, or high concentrations as defined above. Cells were harvested at 12 h for analysis of CEA (CEACAM5) mRNA expression, and culture supernatants were collected at 48 h for measurement of secreted CEA protein. CEA mRNA levels were quantified by RT–qPCR using GAPDH as the reference gene, and secreted CEA concentrations were determined using a human CEA ELISA kit (Abcam, ab99992). Samples were diluted when necessary to fall within the linear range of the standard curve and were analyzed in duplicate.

### Generation of THP-1 conditioned medium and CRC stimulation

To generate conditioned medium, THP-1 cells were treated with vehicle or increasing concentrations of SiO_2_ as described above. After 24 h, culture supernatants were collected and sequentially clarified by centrifugation at 300 × g for 5 min followed by 2,000 × g for 10 min, and then filtered through a 0.22 μm sterile filter to remove residual cells, debris, and particles. Conditioned media were aliquoted and stored at −80 °C. HT29 and Caco-2 cells were stimulated with conditioned media using a fixed ratio of 50% conditioned medium and 50% fresh complete medium (final 10% FBS) to ensure equivalent nutrient and serum conditions across treatment groups. CEA mRNA expression was assessed at 12 h, and secreted CEA protein levels were measured at 48 h.

### Causal validation by suppression of monocyte inflammation and IL-6 neutralization

To establish causality, conditioned media were generated from THP-1 cells exposed to high-dose SiO_2_ in the presence or absence of BAY 11-7082, as well as from cells treated with BAY 11–7082 alone. Conditioned media were collected at 24 h and processed identically to those described above. IL-6 concentrations in conditioned media were quantified by ELISA to confirm effective suppression of inflammatory output following NF-κB inhibition.

CRC cells were then stimulated with conditioned media in the presence or absence of anti-IL-6 antibody or isotype control IgG, which were added at the onset of stimulation and maintained throughout incubation. CEA mRNA expression and protein secretion were analyzed at 12 h and 48 h, respectively.

### Identification of core genes

Core genes associated with SiO_2_ exposure were retrieved from the Comparative Toxicogenomics Database (CTD), while colorectal cancer (CRC)–related core genes were obtained from the GeneCards database. The overlapping genes were defined as potential molecular mediators linking environmental exposure to tumor pathogenesis.

### Transcriptomic validation using GEO and TCGA datasets

Transcriptomic profiles and matched clinical annotations for ovarian cancer were obtained from The TCGA and GEO. Only samples with complete follow-up information were included in the analysis. Genes with a prioritization score greater than four from the preceding integrative analysis were selected for clinical correlation assessment. Survival endpoints included overall survival (CRC) in both TCGA and GEO. For each candidate gene, patients were stratified into high- and low-expression groups based on the median expression value within each cohort. Univariate survival analysis was performed using the Kaplan–Meier method with the log-rank test, while multivariable Cox proportional hazards regression models were applied to estimate hazard ratios (HRs) and 95% confidence intervals (CIs), adjusting for relevant clinical covariates. Genes demonstrating significant survival associations were subjected to functional enrichment analysis.

### Single-cell RNA sequencing analysis

The preprocessing and analysis of the single-cell RNA sequencing dataset GSE161277 were supported by the KeyanZhiJia Single-Cell Analysis Platform.

### Statistical analysis

Data are presented as mean ± standard deviation (SD). Statistical analyses were performed using GraphPad Prism 9.0. One-way analysis of variance (ANOVA) followed by Dunnett’s or Tukey’s *post hoc* tests was applied as appropriate. Normality and homogeneity of variance were assessed using the Shapiro–Wilk and Levene tests, respectively, and data were log-transformed when necessary. A two-sided P < 0.05 was considered statistically significant.

## Results

### Performance comparison of machine learning models

To systematically evaluate the robustness and applicability of different machine learning paradigms in predicting carcinoembryonic antigen (CEA) positivity, we constructed and compared 14 representative algorithms across major method families. These include tree based ensemble models (random forest, gradient enhancement, adaptive enhancement, LightGBM, and CatBoost), regression based methods (logistic regression, LASSO, and partial least squares), kernel and distance based methods (support vector machines and k-nearest neighbors), probability models (naive Bayes and discriminant analysis), and neural networks (multilayer perceptron). The dataset is randomly divided into training (70%) and testing (30%) subsets, and hyperparameters are optimized using five fold cross validation. The model performance was evaluated in multiple dimensions, including discriminative ability, predictive consistency, and potential clinical utility.

**Figure 1:**
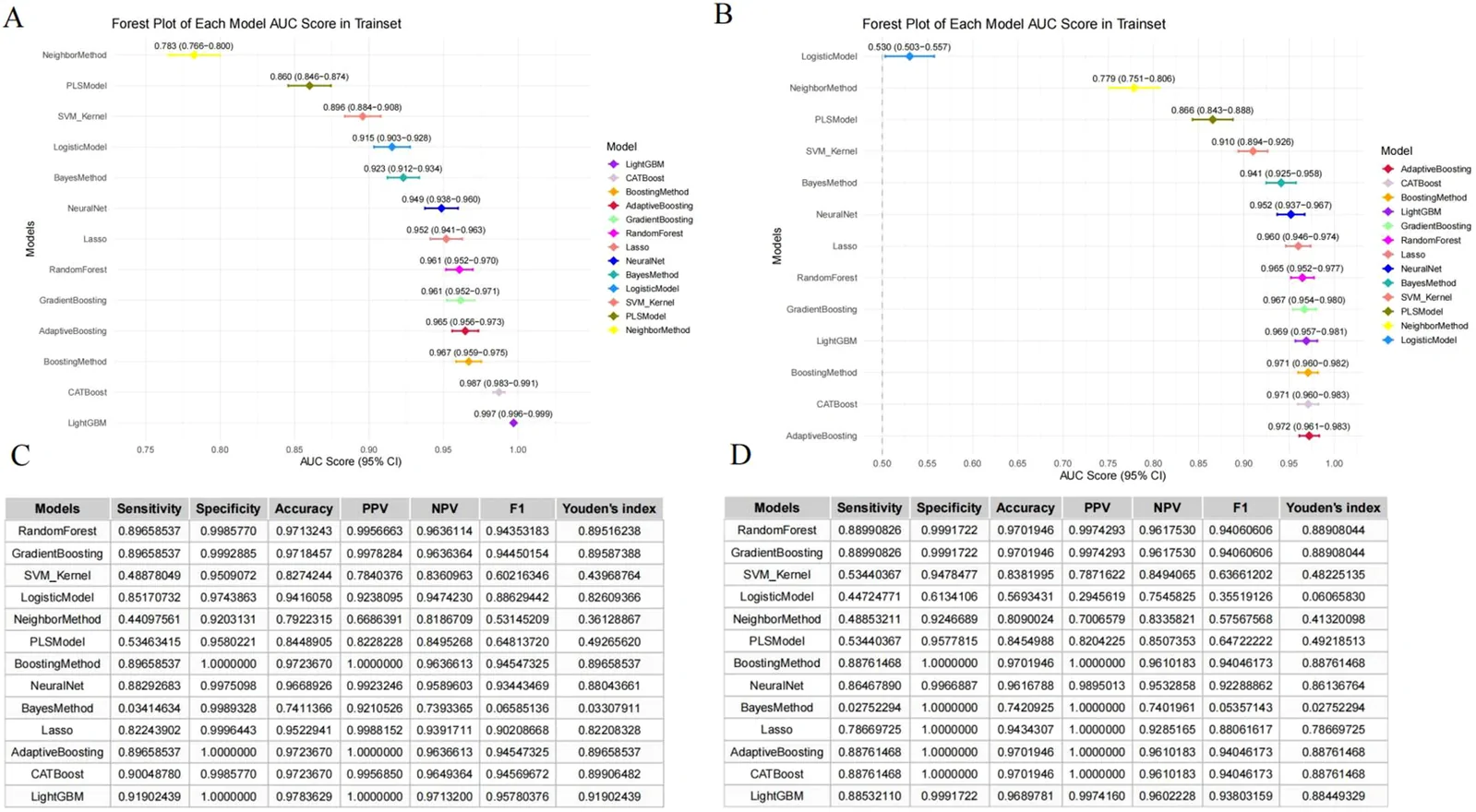
Performance comparison of machine learning models for predicting CEA positivity. **(A)** Forest plot of AUC scores (with 95% confidence intervals) for each model in the training set. **(B)** Forest plot of AUC scores (with 95% confidence intervals) for each model in the testing set. **(C)** Performance metrics of all models in the training set, including sensitivity, specificity, accuracy, positive predictive value (PPV), negative predictive value (NPV), F1 score, and Youden’s index. **(D)** Performance metrics of all models in the testing set, including sensitivity, specificity, accuracy, positive predictive value (PPV), negative predictive value (NPV), F1 score, and Youden’s index.

Clear method stratification has emerged in data segmentation and evaluation metrics. Set based models consistently outperform regression, kernel, and distance based methods in both training and testing sets. During the training phase, LightGBM and CatBoost achieved near saturation discriminative performance with AUC values close to 1.00, followed by adaptive enhancement, gradient enhancement, and random forest. In contrast, logistic regression, k-nearest neighbor, and naive Bayes showed significantly weaker discriminative power, highlighting the limited expressive ability of simpler models in capturing complex exposure biomarker relationships (**Figures 1A, B**). It is worth noting that the performance differences in the test set have become more pronounced. CatBoost maintains excellent sensitivity, specificity, F1 score, and overall accuracy, with minimal degradation relative to training performance, indicating its strong generalization ability and stable classification behavior (**Figures 1C, D**). These findings indicate that ensemble learning frameworks not only perform well in model fitting, but also have structural robustness in out of sample prediction.

**Figure 2:**
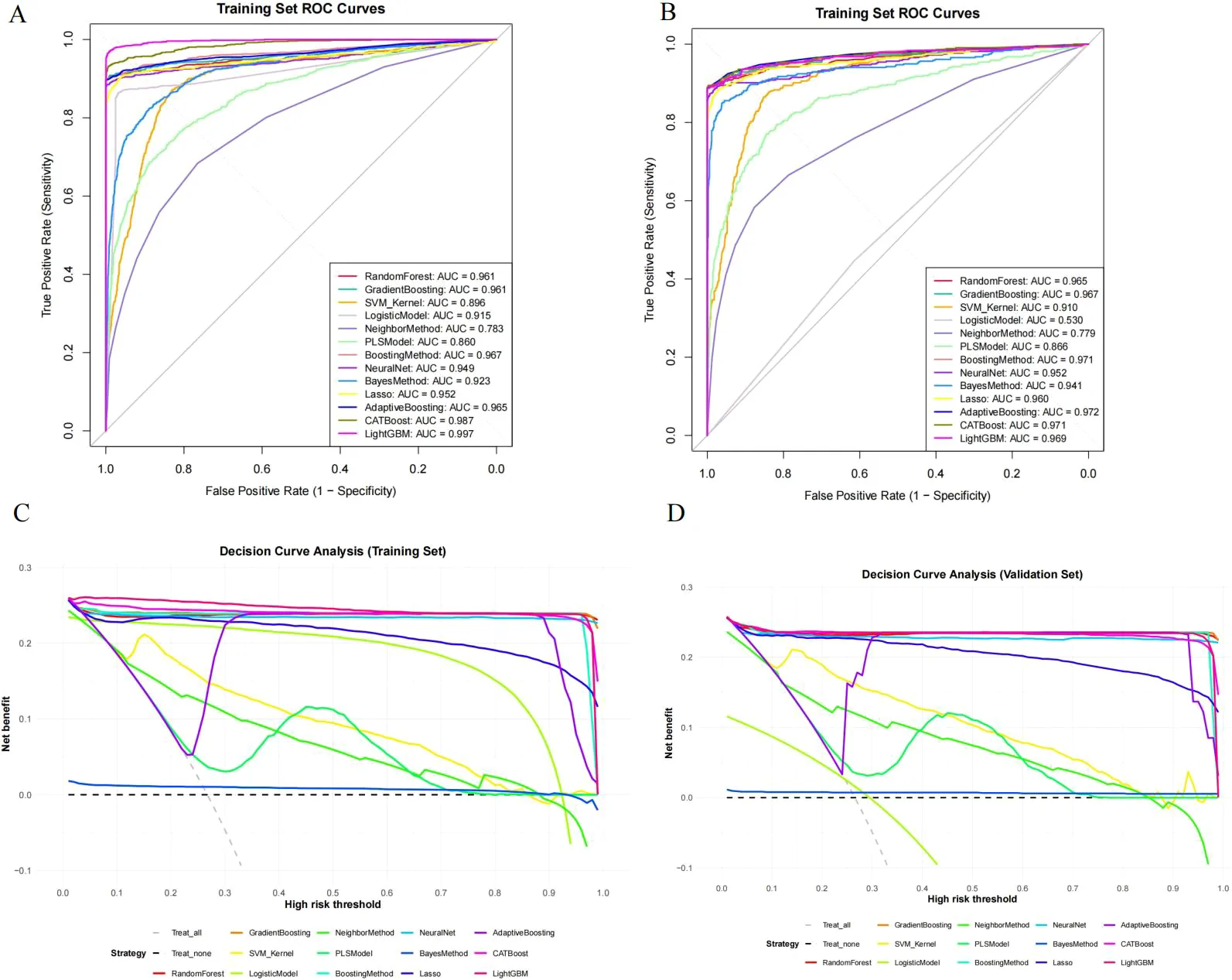
Model discrimination and clinical utility across training and testing datasets. **(A)** ROC curves of all models in the training set. **(B)** ROC curves of all models in the testing set. **(C)** Decision curve analysis (DCA) for each model in the training set. **(D)** Decision curve analysis (DCA) for each model in the testing set.

**Figure 3:**
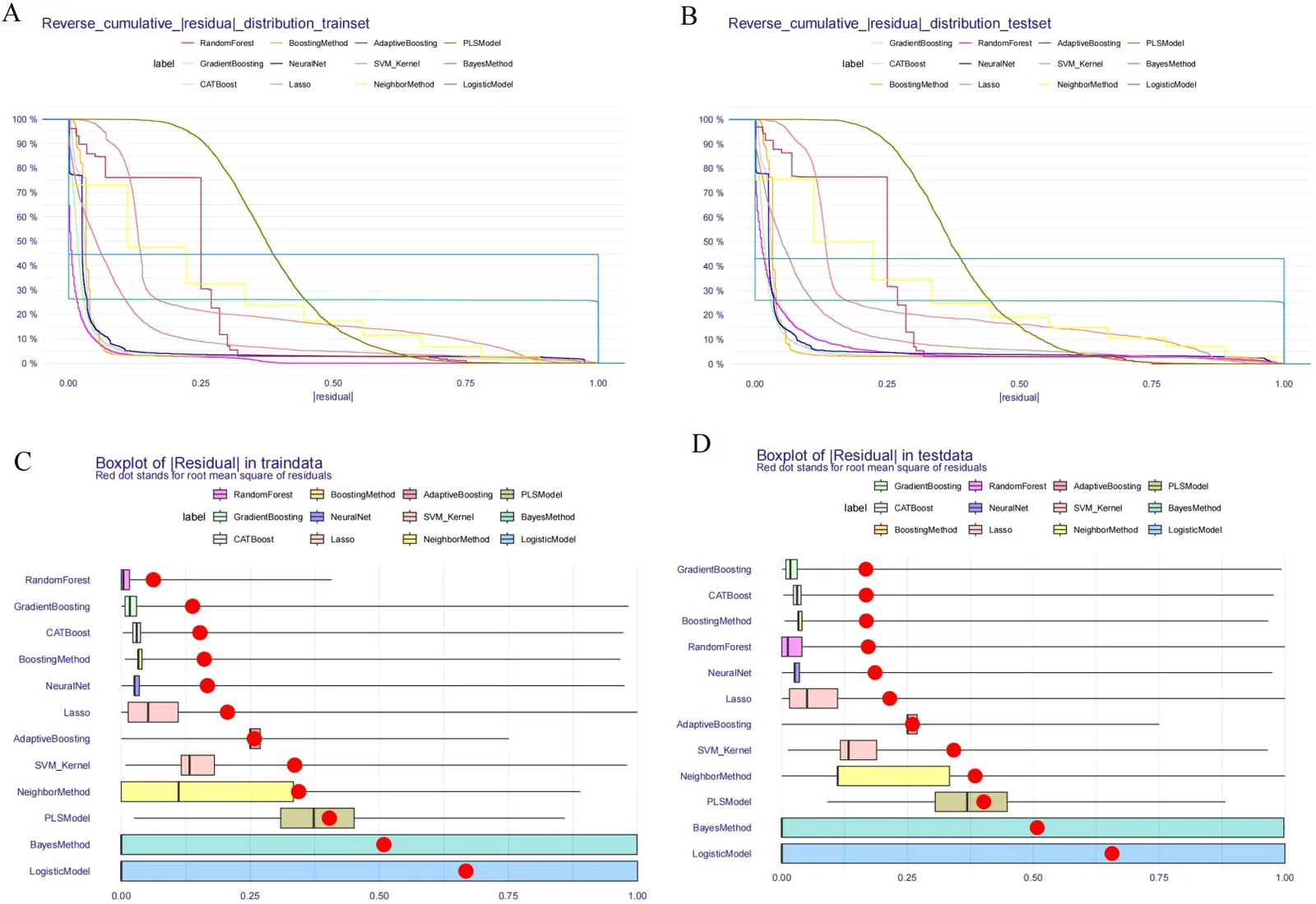
Residual distribution analysis across training and testing sets. **(A)** Reverse cumulative distribution of absolute residuals for each model in the training set. **(B)** Reverse cumulative distribution of absolute residuals for each model in the testing set. **(C)** Boxplot of residuals in the training set. Red dots indicate root mean square of residuals for each model. **(D)** Boxplot of residuals in the testing set. Red dots indicate root mean square of residuals for each model.

The analysis of receiver operating characteristics further confirms these trends (**Figures 2A, B**). In the training set, CatBoost and LightGBM have consistently dominated the ROC space, while in the test set, CatBoost exhibits the smallest curve collapse, highlighting its resilience to sample perturbations (**Figures 2A, B**). However, relying solely on discriminative performance cannot fully capture the reliability of the model. To address this issue, DCA was used to evaluate potential clinical relevance (**Figures 2C, D**). Within a wide range of threshold probabilities, CatBoost achieved the highest net benefit in both training and testing queues, outperforming alternative algorithms and default strategies (“treat all” and “do not treat”) (**Figures 2C, D**). This indicates that CatBoost provides excellent decision level value for identifying high-risk individuals with CEA positivity, supporting its translational relevance to occupational risk stratification (**Figures 3A, B**). The stability of the model was further verified through residual structure analysis. The reverse cumulative residual distribution indicates that CatBoost consistently exhibits a steep left shift curve in both the training and testing sets, reflecting a significant reduction in the proportion of large prediction errors. Correspondingly, the residual boxplot RMSE of CatBoost, while logistic regression, naive Bayes, and partial least squares models show significant long tail error distributions (**Figures 3C, D**). Overall, these analyses indicate that the model’s advantage depends not only on average performance metrics, but also on controlled and reproducible erroneous behavior.

### Model interpretation using SHAP values

**Figure 4:**
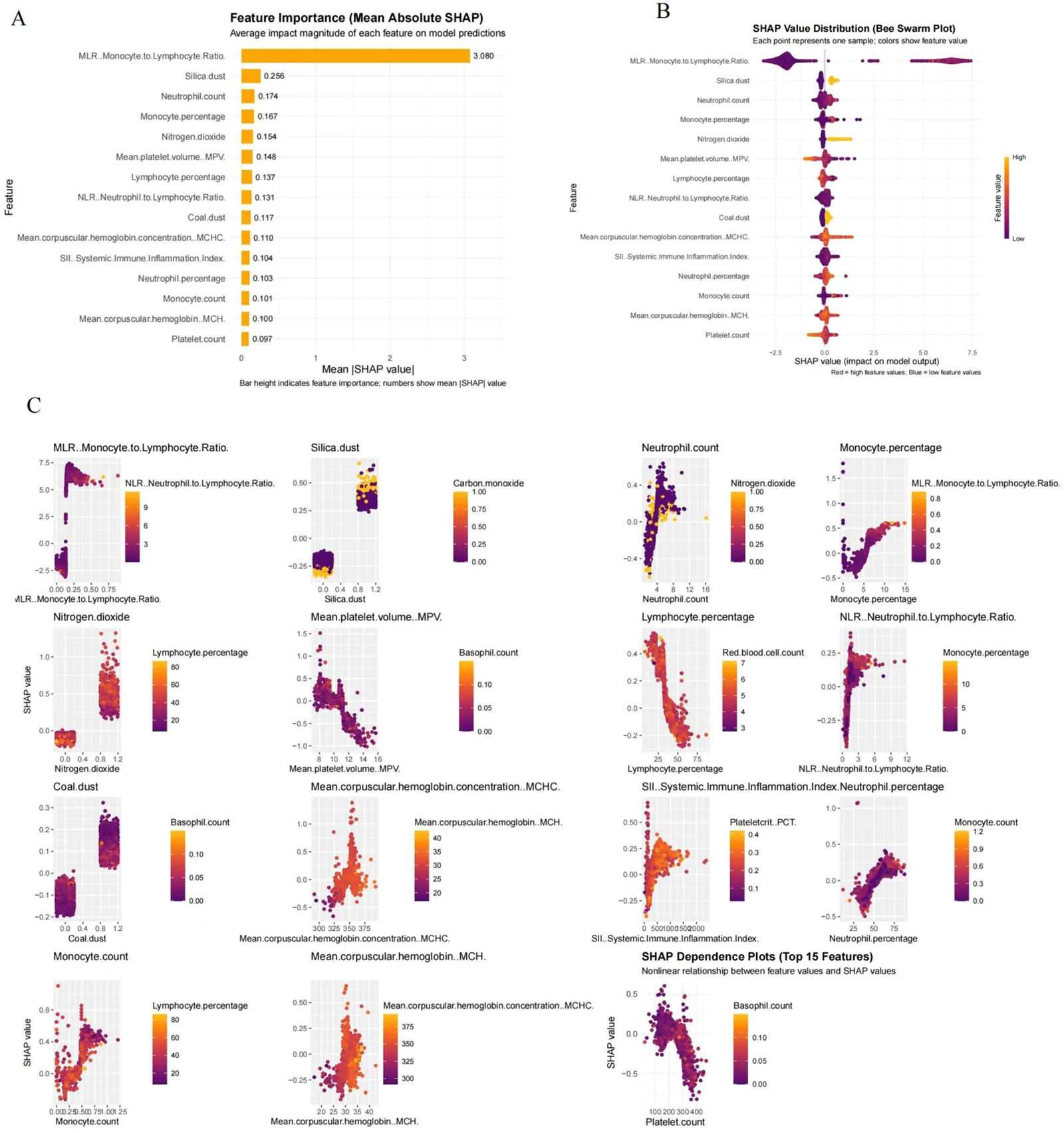
SHAP-based interpretation of model predictions. **(A)** Feature importance ranked by mean absolute SHAP values. **(B)** SHAP beeswarm plot showing the distribution and impact direction of SHAP values across all samples. Each point represents a single observation; color indicates the corresponding feature value. **(C)** SHAP dependence plots of the top 15 most important features. Each subplot shows the non-linear relationship between the feature value and its SHAP contribution to the model output. Color gradients indicate possible interactions with secondary features.

Among the 14 machine learning algorithms evaluated, CatBoost demonstrated the best overall performance on the test set in terms of AUC, sensitivity, and specificity. In addition to its strong predictive ability, CatBoost is particularly well-suited for handling complex nonlinear relationships and categorical variables without extensive preprocessing. Its high stability and compatibility with SHAP values for feature attribution made it a practical and interpretable choice for this real-world dataset. The feature attribution using the SHAP framework indicates that CEA positivity is mainly driven by hematological and inflammation related variables, with MLR being the most influential predictor (**Figures 4A, B**). This discovery emphasizes that systemic immune imbalance is the core determinant of CEA abnormal expression. Among all occupational and environmental exposures, the SHAP contribution of silica dust is the highest, significantly higher than that of nitrogen dioxide, coal dust, and carbon monoxide. SHAP distribution and dependency analysis further indicate that the association between silica exposure and CEA positivity is clearly nonlinear, characterized by threshold like effects and significant inter individual heterogeneity at higher exposure levels (**Figure 4C**). These patterns indicate that silicon dust may act as a risk amplifier rather than producing uniform linear effects.

### Mediation analysis of inflammatory markers

**Figure 5:**
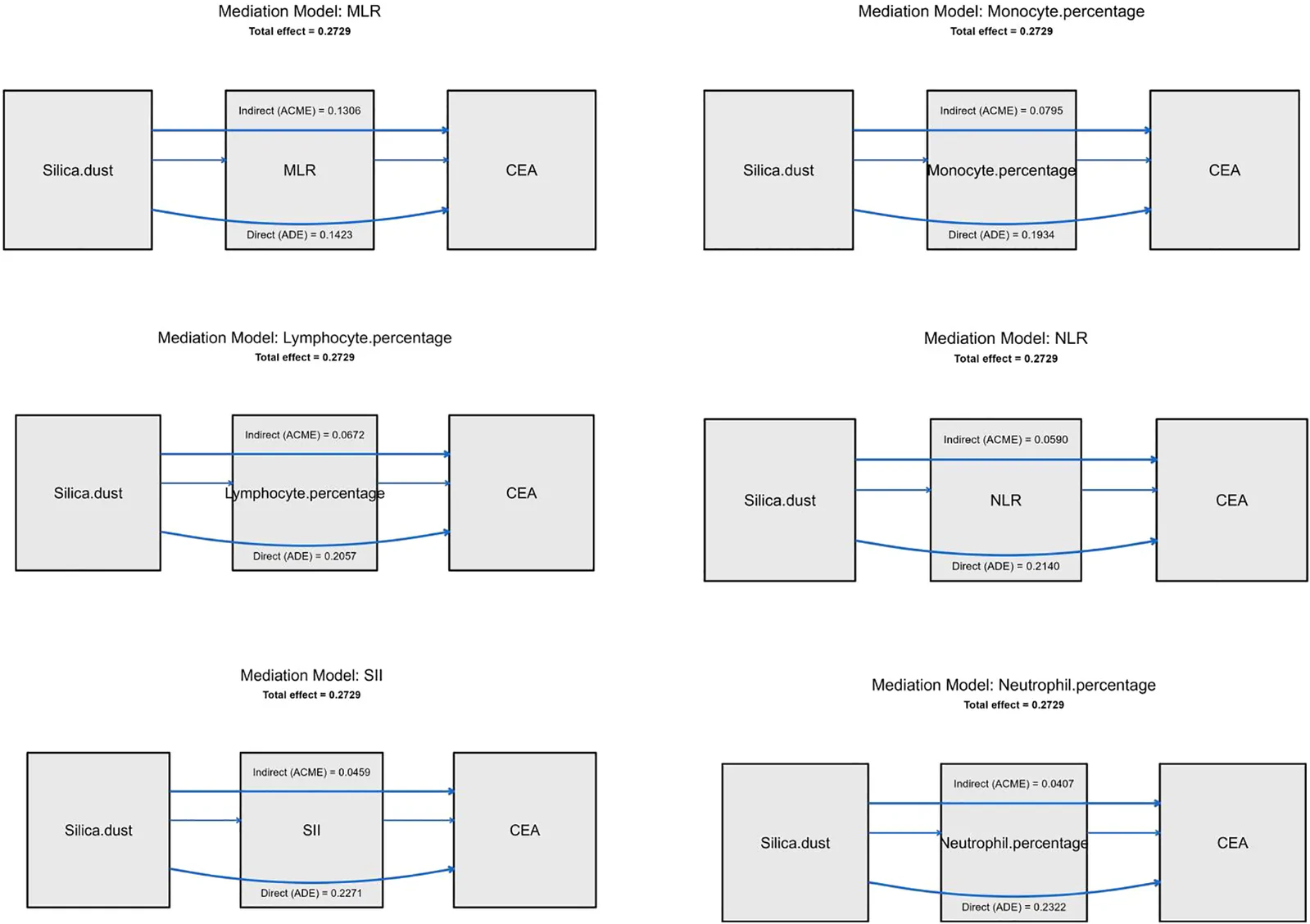
Mediation analysis of inflammatory indices linking silica dust exposure and CEA elevation.

To elucidate the potential biological pathways behind this structural association, mediation analysis was conducted to evaluate the role of inflammatory markers in linking silica exposure to CEA elevation (**Figure 5**). Multiple biomarkers including MLR, neutrophil percentage, lymphocyte percentage, NLR, and SII showed significant indirect effects, indicating that the relationship between silica and CEA is partially mediated through the inflammatory pathway. It is worth noting that MLR accounts for the largest proportion in mediating effects, supporting a model in which monocyte dominated inflammatory activation represents a key mechanistic link between occupational silica exposure and elevated CEA levels.

### *In vitro* validation of silica-induced cytokine responses

**Figure 6:**
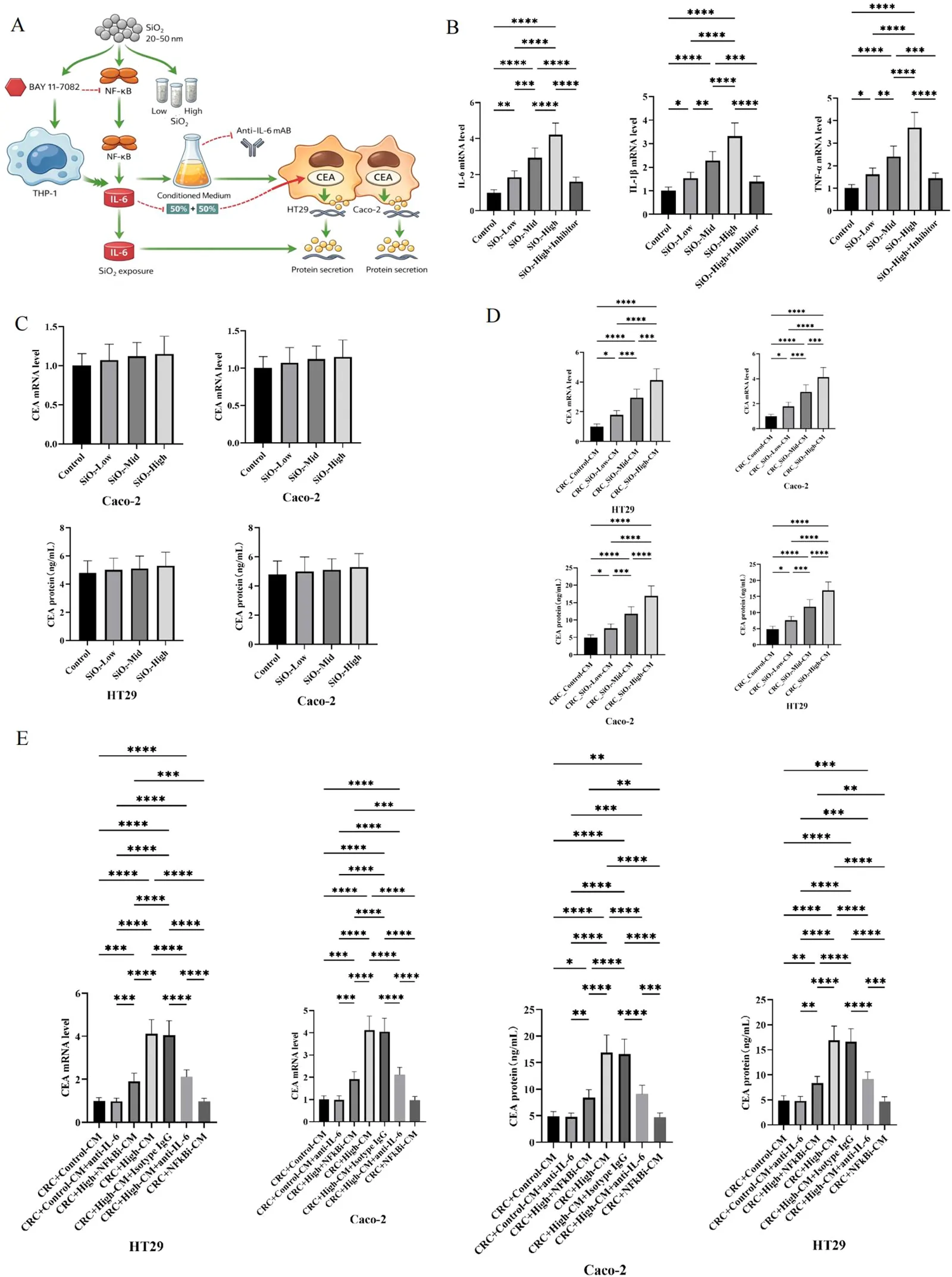
*In vitro* evaluation of monocyte-derived cytokine signaling on CEA expression in colorectal cancer cells. **(A)** Schematic diagram of the experimental workflow. THP-1 monocytes were exposed to amorphous silica (SiO_2_) and treated with NF-κB inhibitor (BAY 11-7082) or IL-6 neutralizing antibody. Conditioned media (CM) were collected and applied to colorectal cancer cell lines (HT29 and Caco-2) to evaluate CEA mRNA and protein expression. **(B)** RT-qPCR quantification of IL-6, IL-1β, and TNF-α mRNA expression in THP-1 cells following low/high-dose SiO_2_ stimulation with or without BAY 11–7082 treatment. **(C)** CEA mRNA and protein levels in HT29 and Caco-2 cells directly treated with SiO_2_ particles (without conditioned media exposure). **(D)** CEA mRNA and protein expression in HT29 and Caco-2 cells treated with conditioned media derived from silica-stimulated THP-1 cells, with or without IL-6 neutralization or NF-κB inhibition. **(E)** CEA mRNA and protein levels in colorectal cancer cells treated with CM under varying conditions: anti-IL-6 antibody, NF-κB inhibitor (BAY 11-7082), or both. Statistical comparisons are shown between multiple experimental groups. Significance levels: P < 0.05 (*), P < 0.01 (**), P < 0.001 (***), P < 0.0001 (****).

As shown in **Figures 6A, B**, SiO_2_ exposure strongly activates THP-1 monocytes in a dose-dependent manner. The mRNA levels of IL-6, IL-1 β, and TNF - α gradually increased from the low-dose and medium dose groups to the high-dose group, and multiple pairwise comparisons reached statistical significance (asterisk annotation). Importantly, the addition of inhibitors under high-dose conditions significantly weakened the induction of transcription of all three cytokines, restoring expression to the low-dose range. These findings indicate that SiO _2_ - induced monocyte activation is inhibitor sensitive and regulated, rather than reflecting non-specific transcriptional fluctuations. In contrast, direct exposure of CRC cells to SiO _2_ only resulted in minor changes in CEA expression (**Figure 6C**). In Caco-2 and HT29 cells, CEA mRNA levels remained close to baseline at low, medium, and high SiO2 levels, and the secreted CEA protein only showed slight upward drift, without a clear dose-response relationship or consistent statistical significance. This pattern supports the explanation that CEA is not strongly regulated by SiO _2_ under these conditions, through direct tumor cell autonomous mechanisms.

We examined how SiO_2_ exposure affects NF-κB p65 activation by conducting Western blot analysis on cells treated with different SiO_2_ concentrations, with and without an NF-κB inhibitor. **Supplementary Figure 1** shows that NF-κB protein levels were baseline in the control group. SiO_2_ treatment led to a concentration-dependent increase in NF-κB expression, with the highest levels in the SiO_2_-High group, followed by the SiO_2_-Mild and SiO_2_-Low groups, all significantly higher than the control (P < 0.0001). The NF-κB p65 inhibitor significantly reduced NF-κB p65 upregulation in the SiO_2_-High+Inhibitor group (P < 0.01), while the inhibitor alone did not affect basal NF-κB p65 levels. The results suggest that exposure to SiO_2_ triggers the NF-κB pathway based on concentration, and this activation can be significantly reduced by using an NF-κB inhibitor.

It is noteworthy that CM derived from SiO _2_ - stimulated THP-1 cells induced significant and graded CEA induction in CRC cells (**Figure 6D**). In Caco-2 and HT29, CEA mRNA gradually increases with the CM produced by low, medium, and high doses of SiO2. The parallel increase of CEA protein secreted by treated monocytes reaches its maximum level under high CM conditions. The consistent stepwise pattern observed at the transcript and protein levels, as well as dense significant annotations, indicate that the soluble factors released by activated monocytes are sufficient to drive strong CEA responses in CRC cells, and the intensity of this response is proportional to the strength of upstream monocyte activation.

Finally, the intervention experiment supported the causal interruption of the paracrine axis (**Figure 6E**). High CM produced the strongest increase in CEA mRNA and protein in two CRC cell lines, while the isotype IgG control group remained comparable to the high CM group, indicating that antibody addition alone cannot explain the observed effects. In contrast, CM (high+NFkBi CM) produced under NF - κ B inhibition significantly reduced the increase in CEA driven by high CM, and the neutralization of IL-6 during CRC stimulation also weakened the expression of CEA at the mRNA and protein levels. It is worth noting that in the context of CM control, anti-IL-6 therapy is still close to baseline, supporting pathway specificity and opposing the main direct inhibitory effect of antibody therapy under non inflammatory conditions.

Overall, this set of numbers supports a coherent mechanistic model, in which SiO_2_ dose-dependent activates THP-1 inflammatory signaling, direct SiO_2_ exposure has little effect on CRC CEA, and monocyte derived conditioned medium can effectively induce CEA in CRC cells in a dose-dependent manner. Partial reversal of NF - κ B inhibition (upstream, monocyte side) and IL-6 neutralization (downstream) suggests that this inflammatory paracrine pathway is partially but not exclusively necessary, consistent with the multifactorial monocyte secretion group driving CRC CEA upregulation.

### Identification of overlapping genes and prognostic modeling

**Figure 7:**
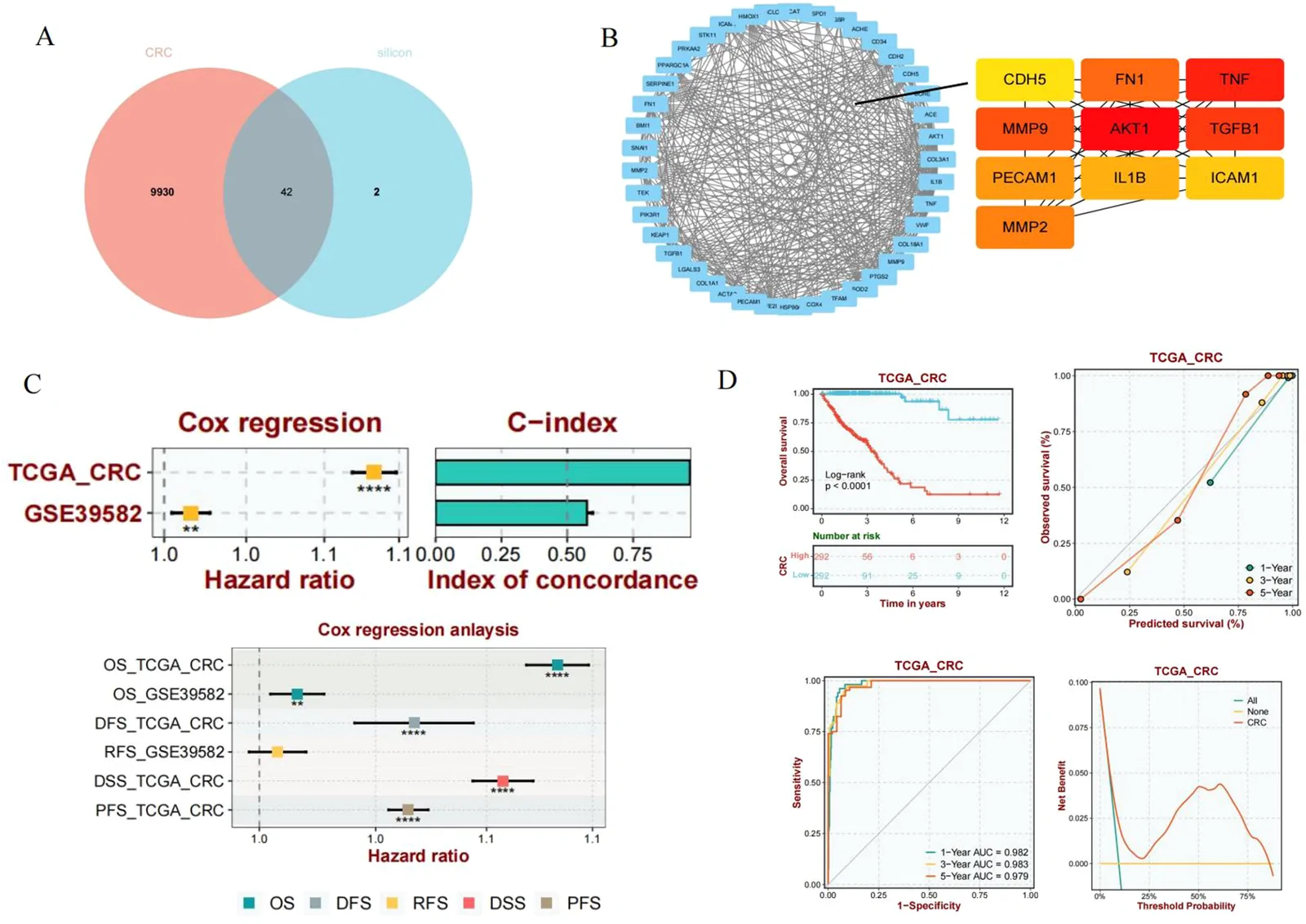
Identification of shared core genes between SiO_2_ exposure and colorectal cancer and their prognostic significance. **(A)** Venn diagram showing the overlap between SiO_2_-associated genes derived from the CTD database and CRC-related genes obtained from GeneCards. A total of 42 overlapping genes were identified. **(B)** Protein–protein interaction (PPI) network constructed using the overlapping genes. Key hub genes with high connectivity are highlighted. **(C)** Multivariable Cox regression analysis and concordance index (C-index) evaluating the prognostic performance of the core gene signature in the TCGA-CRC and GSE39582 cohorts across multiple survival endpoints. **(D)** Prognostic evaluation of the gene signature in the TCGA-CRC cohort. Kaplan–Meier survival curves comparing high- and low-risk groups, time-dependent ROC curves for 1-, 3-, and 5-year overall survival, and decision curve analysis (DCA) illustrating the clinical net benefit of the model. **, **** P< 0.01, P<0.0001.

SiO_2_-associated genes were retrieved from the Comparative Toxicogenomics Database (CTD), while CRC–related genes were obtained from the GeneCards database. An intersection analysis identified a total of 42 overlapping genes that were simultaneously associated with SiO_2_ exposure and CRC (**Figure 7A**), suggesting that these genes may serve as potential molecular bridges linking environmental exposure to tumorigenesis. To further characterize the functional relationships among the overlapping genes, a protein–protein interaction (PPI) network was constructed (**Figure 7B**). Network analysis revealed a highly interconnected interaction pattern among these genes, indicating their involvement in shared biological processes. Based on network topological features, a set of key hub genes was identified, including AKT1, TNF, TGFB1, IL1B, MMP9, MMP2, FN1, ICAM1, PECAM1, and CDH5. These genes exhibited high connectivity within the network and are likely to play central roles in regulating inflammatory responses, extracellular matrix remodeling, and endothelial function.

A risk score model was constructed based on the identified core genes and evaluated in the TCGA-CRC and GSE39582 cohorts to assess its prognostic significance. Multivariable Cox regression analysis demonstrated that the gene signature was significantly associated with overall survival (OS) (**Figure 7C**) and consistently exhibited robust prognostic performance across both independent cohorts. The concordance index (C-index) further confirmed the predictive accuracy of the model. Kaplan–Meier survival analysis in the TCGA-CRC cohort showed that patients in the high-risk group had significantly poorer overall survival compared with those in the low-risk group (log-rank P < 0.0001) (**Figure 7D**). Time-dependent receiver operating characteristic (ROC) curves indicated strong discriminatory power for 1-, 3-, and 5-year survival prediction. In addition, decision curve analysis (DCA) demonstrated that the risk model provided a stable net clinical benefit across a wide range of threshold probabilities, highlighting its potential clinical utility.

### Single-cell transcriptomic insights into tumor microenvironment remodeling

**Figure 8:**
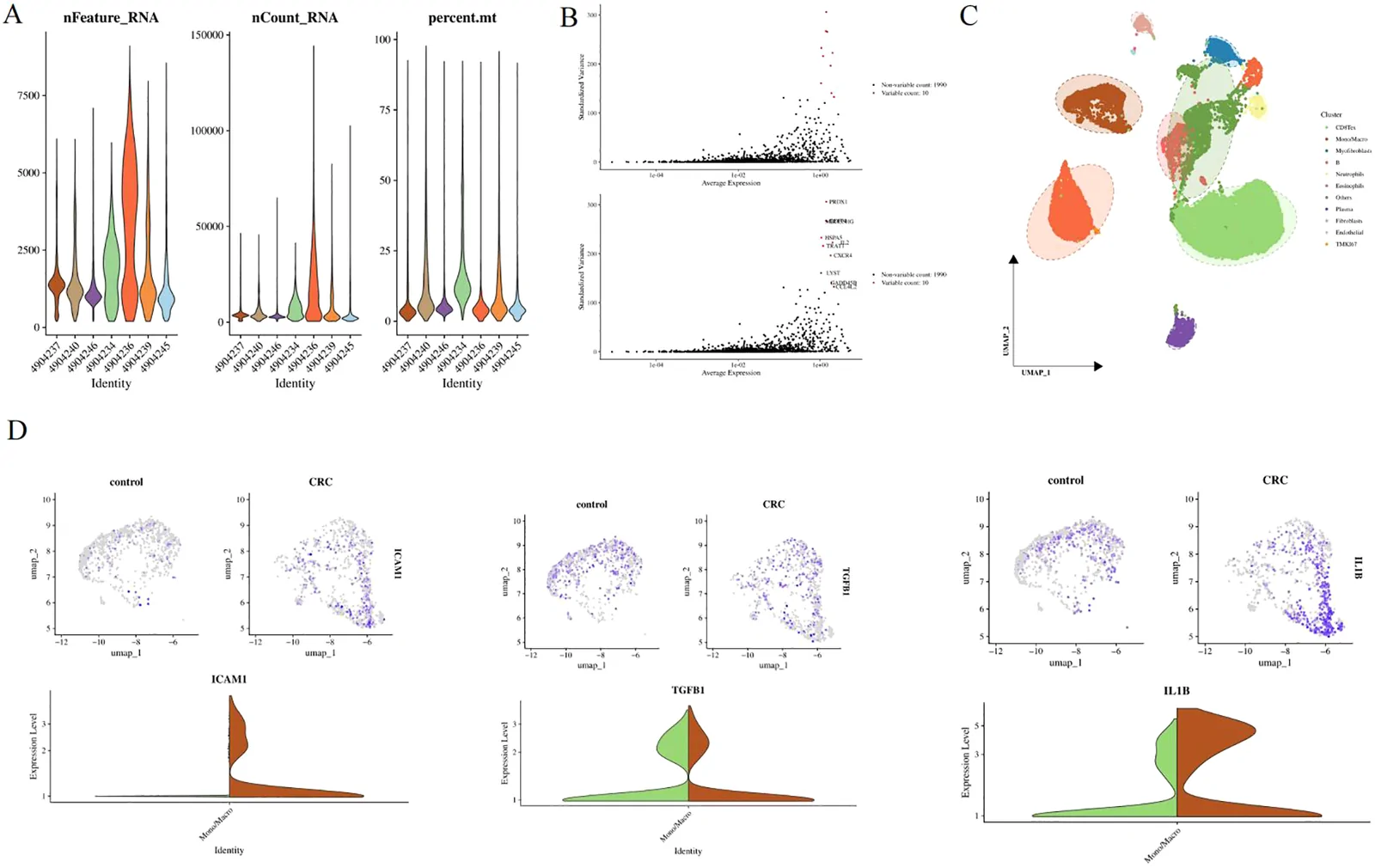
Single-cell transcriptomic evidence linking immune dysregulation to tumor microenvironment remodeling in colorectal cancer. **(A)** Quality control metrics of single-cell RNA sequencing data from the GSE161277 dataset, showing the distributions of detected genes (nFeature_RNA), total UMI counts (nCount_RNA), and mitochondrial gene proportions (percent.mt) across samples. **(B)** Identification of highly variable genes following normalization and scaling, capturing dominant biological heterogeneity for downstream analysis. **(C)** Uniform Manifold Approximation and Projection (UMAP) visualization of single cells colored by annotated cell types based on canonical marker genes, including immune and stromal populations such as T cells, B cells, monocytes, macrophages, neutrophils, fibroblasts, endothelial cells, and epithelial cells. **(D)** Feature plots and violin plots showing the expression patterns of inflammation- and signaling-related genes (ICAM1, TGFB1, and IL1B) in control and CRC samples.

To provide cell-level evidence supporting the role of SiO_2_ in shaping tumor microenvironment susceptibility, we analyzed single-cell RNA sequencing data from the GSE161277 dataset. After stringent quality control, the cells retained for downstream analysis exhibited comparable distributions of detected genes (nFeature_RNA), total UMI counts (nCount_RNA), and mitochondrial gene proportions (percent.mt) across samples (**Figure 8A**), indicating high data quality and minimal technical bias. Following normalization and dimensionality reduction, highly variable genes were identified to capture dominant biological heterogeneity (**Figure 8B**). Unsupervised clustering combined with Uniform Manifold Approximation and Projection (UMAP) visualization revealed well-separated cellular populations (**Figure 8C**). Based on canonical marker genes, these clusters were annotated as major immune and stromal cell types, including T cells, B cells, monocytes, macrophages, neutrophils, fibroblasts, endothelial cells, and epithelial cells, reflecting the complex cellular architecture of the colorectal tumor microenvironment. To directly interrogate inflammation-related transcriptional alterations associated with occupational exposure–related immune imbalance, we examined the expression patterns of key genes involved in immune activation, cytokine signaling, and cell–cell interactions. Comparative analysis between control and CRC samples demonstrated marked upregulation of ICAM1, TGFB1, and IL1B in CRC-derived cells (**Figure 8D**). Feature plots revealed that these genes were enriched within specific immune and stromal compartments rather than being uniformly expressed, indicating localized microenvironmental activation rather than global transcriptional shifts. Violin plot analysis further confirmed significantly higher expression levels of these genes in CRC samples compared with controls.

Collectively, these single-cell results provide direct cellular evidence that colorectal cancer is characterized by coordinated immune and stromal transcriptional reprogramming, consistent with a state of sustained inflammatory activation. The enrichment of adhesion molecules and cytokine-related genes supports a model in which systemic immune dysregulation—potentially triggered by chronic occupational exposure—contributes to tumor microenvironment remodeling, thereby creating a permissive context for tumor-associated biomarker elevation and disease progression.

## Discussion

Our research findings collectively depict a mechanism and computational framework linking occupational silica exposure to elevated levels of carcinoembryonic antigen (CEA) through inflammation mediated pathways. By combining real-world occupational health monitoring data with machine learning and *in vitro* validation, we provide multidimensional evidence to support CEA as a reactive biomarker that reflects environmental exposure and promotes tumor microenvironment remodeling.

From a computational perspective, the outstanding performance of ensemble learning models highlights the importance of capturing nonlinear, multidimensional interactions between occupational exposure, hematological parameters, and systemic inflammation indices. The interpretability of SHAP based models further reveals inflammation related features, especially MLR, which is a key mediator linking specific occupational toxins with elevated CEA. These findings extend previous reports that inflammatory features may occur prior to obvious malignant tumors, emphasizing the potential of artificial intelligence driven frameworks in exposure risk stratification (22, 23). At the biological level, our *in vitro* experiments confirmed that amorphous silica particles induce strong monocyte activation characterized by NF - κ B-dependent IL-6 secretion. The conditioned medium derived from activated monocytes significantly increased CEA expression in colorectal cancer cells, partially reversed after drug inhibition or cytokine neutralization. These results suggest that elevated CEA levels in exposed workers may reflect chronic immune disorders, rather than just cellular autonomous genotoxic effects (24). This mechanism may be particularly relevant to tissues with active epithelial renewal, such as the lungs, colon, or bladder (25). Finally, given that CEA testing is widely accessible, inexpensive, and already incorporated into routine health screening protocols, the ability to predict CEA elevation using machine learning offers a scalable and practical tool for occupational cancer risk stratification, enabling proactive surveillance and early intervention in exposed populations.

Mechanistically, the pathway linking silica exposure to CEA upregulation involves a multi-step cascade centered around monocyte activation and paracrine signaling. After inhalation, amorphous silica particles accumulate in the alveolar region, where they are engulfed by resident or recruited monocytes/macrophages. This activates innate immune signaling pathways, especially the NF-κB axis, leading to upregulation and secretion of pro-inflammatory cytokines, including IL-6, TN -α, and IL-1β (26). SO, in our SHAP analysis, monocyte-to-lymphocyte ratio (MLR) and neutrophil count emerged as the most important predictors of CEA positivity. Biologically, these features reflect systemic immune dysregulation consistent with chronic silica exposure. Elevated MLR suggests a shift toward a myeloid-dominant immune profile, which has been associated with macrophage-driven inflammation, cytokine release (e.g., IL-6), and tumor-promoting paracrine signaling. Increased neutrophil counts may reflect acute-phase responses and have been implicated in tumor progression through reactive oxygen species generation, immune suppression, and neutrophil extracellular trap (NET) formation. These patterns align with recent studies showing that silica inhalation promotes myeloid expansion, NF-κB activation, and inflammatory remodeling of the tumor microenvironment (26). Therefore, the top SHAP features not only carry predictive power, but also biological relevance in the context of exposure-related carcinogenesis.

IL-6 is a pleiotropic cytokine that plays a dual role in inflammation and cancer biology. In our study, IL-6 served as a key soluble mediator linking monocyte activation with tumor cell responses. When CRC cells were exposed to conditioned medium containing IL-6 (possibly other co releasing factors), a significant increase in CEA mRNA expression and protein secretion was observed. The neutralizing effect of IL-6 weakened this effect, but it was not completely eliminated, indicating that although IL-6 is necessary, there may be other mediators at work (24). Importantly, the response of CRC cells to monocyte derived signals is not only a passive response, but also involves reprogramming of the transcriptional network that controls the expression of tumor biomarkers. It is known that CEA (CEACAM5) is upregulated in the context of inflammatory cytokine exposure, and its promoter region contains binding sites that respond to downstream effector STAT3 of IL-6 signaling (27). Therefore, sustained exposure to IL-6 may activate the JAK/STAT3 pathway, leading epithelial cells to develop a pro tumor phenotype, enhancing immune evasion, cell adhesion, and potentially increasing metastatic potential. In addition, incomplete reversal of CEA induction by NF - κ B blockade in monocytes or IL-6 neutralization in tumor cells indicates redundancy and complexity in the inflammatory secretion group. Other mediators, such as IL-8, GM-CSF, or ROS, may synergistically interact with IL-6 or act in parallel pathways to shape TME. This multifactorial inflammatory environment may become a potential risk amplifier, especially in chronic or cumulative exposure situations (28). In addition to IL-6, we also observed marked mRNA upregulation of IL-1β and TNF-α upon silica stimulation; however, their protein levels were not assessed in the current study. This represents a limitation of our cytokine profiling, and we acknowledge that these additional secreted factors may contribute to downstream effects. Further protein-level validation is warranted to fully characterize the inflammatory secretome of exposed monocytes.

These findings reinforce the emerging understanding that occupational silica exposure not only triggers local inflammation but also reprograms immune cell function, contributing to early TME susceptibility. Recent studies have shown that amorphous and crystalline forms of silica activate macrophages through NF-κB and NLRP3 pathways, leading to sustained secretion of IL-6 and other proinflammatory cytokines (29–32). Our *in vitro* results further support this model, demonstrating that THP-1–derived conditioned media can upregulate CEA in CRC cells via an IL-6–dependent mechanism. In parallel, our scRNA-seq analysis revealed increased expression of inflammation- and adhesion-related genes in tumor-associated macrophages, consistent with prior reports in colorectal cancer cohorts (33). These integrated findings suggest that immune dysregulation may represent a key intermediate link between occupational exposure and early cancer risk, particularly in high-risk working populations.

Our research has several implications. Firstly, incorporating CEA into occupational monitoring programs can promote early identification of high-risk populations. Secondly, using interpretable models can transparently explain the exposure biomarker relationship, strengthen regulatory accountability, and enable personalized risk assessment. Thirdly, identifying inflammation as a key mediator paves the way for intervention strategies targeting the immune microenvironment, including anti-inflammatory or cytokine regulation methods. However, there are several limitations worth considering. The exposure data is based on binary classification obtained from regulatory monitoring, rather than continuous individual dose measurements, which may underestimate the dose-response gradient. And, there were no potentially important confounding variables in this study, such as smoking status, BMI and chronic comorbidities (such as diabetes, cardiovascular disease). The occupational monitoring system used in this study did not regularly collect these data. Although our model includes a variety of hematological and demographic characteristics, unmeasured confounding factors cannot be completely excluded. Secondly, although the focus on silica provides mechanistic clarity, further research is needed to extend it to other carcinogens such as benzene and polycyclic aromatic hydrocarbons. Thirdly, although THP-1 cells are widely used to model macrophage activation in response to environmental stimuli, their leukemic origin may limit generalizability. Future studies incorporating RAW264.7 or PBMC-derived macrophages—used in comparable models of particulate-induced immune activation (29, 30, 34) are warranted to validate the robustness and applicability of our findings. Finally, occupational exposure in this study was classified as a binary variable based on regulatory thresholds, which may not fully capture exposure intensity or duration. And while the model demonstrated good internal performance, external validation in independent populations is necessary to confirm generalizability and robustness. Given the heterogeneity of occupational environments and individual health status, future work should incorporate stratified or sensitivity analyses (e.g., by sex, age group, or job category) to further validate model robustness and generalizability.

In summary, this study supports a model that occupational silica exposure promotes CEA elevation through inflammation driven paracrine signaling. The integration of machine learning, molecular biology, and exposure science provides a powerful paradigm for revealing hidden connections between environmental carcinogens and early risk biomarkers. Future research should extend this method to additional exposures and explore the prognostic value of dynamic CEA monitoring in occupational exposure cohorts.

## Data Availability

The raw data supporting the conclusions of this article will be made available by the authors, without undue reservation.

## Supplementary material

Supplementary Figure 1 NF-κB activation verification.

## References

[1] Marant Micallef C Shield KD Baldi I Charbotel B Fervers B Gilg Soit Ilg A . Occupational exposures and cancer: a review of agents and relative risk estimates. Occup Environ Med. (2018) 75:604–14. doi: 10.1136/oemed-2017-104858 29735747

[2] Wild CP . The exposome: from concept to utility. Int J Epidemiol. (2012) 41:24–32. doi: 10.1093/ije/dyr236 22296988

[3] Rushton L Hutchings SJ Fortunato L Young C Evans GS Brown T . Occupational cancer burden in Great Britain. Br J Cancer. (2012) 107:S3–7. doi: 10.1038/bjc.2012.112 22710676 PMC3384015

[4] Pearce N Blair A Vineis P Ahrens W Andersen A Anto JM . IARC monographs: 40 years of evaluating carcinogenic hazards to humans. Environ Health Perspect. (2015) 123:507–14. doi: 10.1289/ehp.1409149 25712798 PMC4455595

[5] Loomis D Guha N Hall AL Straif K . Identifying occupational carcinogens: an update from the IARC Monographs. Occup Environ Med. (2018) 75:593–603. doi: 10.1136/oemed-2017-104944 29769352 PMC6204931

[6] Straif K Benbrahim-Tallaa L Baan R Grosse Y Secretan B El Ghissassi F . A review of human carcinogens--Part C: metals, arsenic, dusts, and fibres. Lancet Oncol. (2009) 10:453–4. doi: 10.1016/s1470-2045(09)70134-2 19418618

[7] Clapp RW Jacobs MM Loechler EL . Environmental and occupational causes of cancer: new evidence 2005-2007. Rev Environ Health. (2008) 23:1–37. doi: 10.1515/reveh.2008.23.1.1 18557596 PMC2791455

[8] Siemiatycki J Richardson L Straif K Latreille B Lakhani R Campbell S . Listing occupational carcinogens. Environ Health Perspect. (2004) 112:1447–59. doi: 10.1289/ehp.7047 15531427 PMC1247606

[9] Greten FR Grivennikov SI . Inflammation and Cancer: Triggers, Mechanisms, and Consequences. Immunity. (2019) 51:27–41. doi: 10.1016/j.immuni.2019.06.025 31315034 PMC6831096

[10] Landskron G de la Fuente M Thuwajit P Thuwajit C Hermoso MA . Chronic inflammation and cytokines in the tumor microenvironment. J Immunol Res. (2014) 2014:149185. doi: 10.1155/2014/149185 24901008 PMC4036716

[11] Kim SJ Williams D Cheresh P Kamp DW . Asbestos-Induced Gastrointestinal Cancer: An Update. J Gastrointest Dig Syst. (2013) 3:135. doi: 10.4172/2161-069X.1000135 27158561 PMC4856305

[12] Wynn TA Vannella KM . Macrophages in Tissue Repair, Regeneration, and Fibrosis. Immunity. (2016) 44:450–62. doi: 10.1016/j.immuni.2016.02.015 26982353 PMC4794754

[13] Leung CC Yu IT Chen W . Silicosis. Lancet. (9830) 2012:379. doi: 10.1016/S0140-6736(12)60235-9 22534002

[14] Lan Q Zhang L Li G Vermeulen R Weinberg RS Dosemeci M . Hematotoxicity in workers exposed to low levels of benzene. Science. (2004) 306:1774–6. doi: 10.1126/science.1102443 15576619 PMC1256034

[15] Gold P Freedman SO . Demonstration of tumor-specific antigens in human colonic carcinomata by immunological tolerance and absorption techniques. J Exp Med. (1965) 121:439–62. doi: 10.1084/jem.121.3.439 14270243 PMC2137957

[16] Duffy MJ . Carcinoembryonic antigen as a marker for colorectal cancer: is it clinically useful? Clin Chem. (2001) 47:624–30. doi: 10.1093/clinchem/47.4.624 11274010

[17] De Vries BB White SM Knight SJ Regan R Homfray T Young ID . Clinical studies on submicroscopic subtelomeric rearrangements: a checklist. J Med Genet. (2001) 38:145–50. doi: 10.1136/jmg.38.3.145 11238680 PMC1734836

[18] Grivennikov SI Greten FR Karin M . Immunity, inflammation, and cancer. Cell. (2010) 140:883–99. doi: 10.1016/j.cell.2010.01.025 20303878 PMC2866629

[19] Faisal F Danelakis A Bjørk MH Winsvold B Matharu M Nachev P . Prediction of new-onset migraine using clinical-genotypic data from the HUNT Study: a machine learning analysis. J Headache Pain. (2025) 26:70. doi: 10.1186/s10194-025-02014-2 40197205 PMC11977938

[20] Luo Q Hao H Xiwen L Qui X Liu D Liu Y . UBE2D1 as a key biomarker in systemic juvenile idiopathic arthritis: a new perspective on diagnosis and disease activity assessment. Arthritis Res Ther. (2025) 27:140. doi: 10.1186/s13075-025-03606-8 40635084 PMC12239252

[21] Qi X Wang S Fang C Jia J Lin L Yuan T . Machine learning and SHAP value interpretation for predicting comorbidity of cardiovascular disease and cancer with dietary antioxidants. Redox Biol. (2025) 79:103470. doi: 10.1016/j.redox.2024.103470 39700695 PMC11729017

[22] Mantovani A Allavena P Sica A Balkwill F . Cancer-related inflammation. Nature. (2008) 454:436–44. doi: 10.1038/nature07205 18650914

[23] Topol EJ . High-performance medicine: the convergence of human and artificial intelligence. Nat Med. (2019) 25:44–56. doi: 10.1038/s41591-018-0300-7 30617339

[24] Holmer R Wätzig GH Tiwari S Rose-John S Kalthoff H . Interleukin-6 trans-signaling increases the expression of carcinoembryonic antigen-related cell adhesion molecules 5 and 6 in colorectal cancer cells. BMC Cancer. (2015) 15:975. doi: 10.1186/s12885-015-1950-1 26673628 PMC4682226

[25] Blanpain C Fuchs E . Epidermal stem cells of the skin. Annu Rev Cell Dev Biol. (2006) 22:339–73. doi: 10.1146/annurev.cellbio.22.010305.104357 16824012 PMC2405915

[26] Hamilton RF Jr Thakur SA Holian A . Silica binding and toxicity in alveolar macrophages. Free Radic Biol Med. (2008) 44:1246–58. doi: 10.1016/j.freeradbiomed.2007.12.027 18226603 PMC2680955

[27] Lin Y He Z Ye J Liu Z She X Gao X . Progress in Understanding the IL-6/STAT3 Pathway in Colorectal Cancer. Onco Targets Ther. (2020) 13:13023–32. doi: 10.2147/OTT.S278013 33376351 PMC7762435

[28] Hanahan D Weinberg RA . Hallmarks of cancer: the next generation. Cell. (2011) 144:646–74. doi: 10.1016/j.cell.2011.02.013 21376230

[29] Yu R Zhang C Yuan M Ye S Hu T Chen S . Exercise-induced Metabolite N-lactoyl-phenylalanine Ameliorates Colitis by Inhibiting M1 Macrophage Polarization via the Suppression of the NF-κB Signaling Pathway. Cell Mol Gastroenterol Hepatol. (2025) 19:101558. doi: 10.1016/j.jcmgh.2025.101558 40562095 PMC12391280

[30] Yuan M Chen S Lin Z Yu R Chao K Ye S . ACSS2-Mediated Histone H4 Lysine 12 Crotonylation (H4K12cr) Alleviates Colitis via Enhancing Transcription of CLDN7. Adv Sci (Weinh). (2025) 12:e00461. doi: 10.1002/advs.202500461 40650658 PMC12376547

[31] Zhang C Yu R Li S Yuan M Hu T Liu J . KRAS mutation increases histone H3 lysine 9 lactylation (H3K9la) to promote colorectal cancer progression by facilitating cholesterol transporter GRAMD1A expression. Cell Death Differ. (2025) 32:2225–38. doi: 10.1038/s41418-025-01533-4 40707783 PMC12669710

[32] Ke H Li Z Li P Ye S Huang J Hu T . Dynamic heterogeneity of colorectal cancer during progression revealed clinical risk-associated cell types and regulations in single-cell resolution and spatial context. Gastroenterol Rep (Oxf). (2023) 11:goad034. doi: 10.1093/gastro/goad034 37360193 PMC10290555

[33] Liu J Su Y Zhang C Dong H Yu R Yang X . NCOA3 impairs the efficacy of anti-PD-L1 therapy via HSP90α/EZH2/CXCL9 axis in colon cancer. Int Immunopharmacol. (2025) 155:114579. doi: 10.1016/j.intimp.2025.114579 40215778

[34] Erickson GM Finkler SA . Determinants of market share for a hospital’s services. Med Care. (1985) 23:1003–18. doi: 10.1097/00005650-198508000-00008 4021577

