# Supplementary figures and images for: Occupational silica exposure drives systemic immune dysregulation and tumor microenvironment susceptibility: evidence from a real-world study
Source: Front Immunol. 2026 Mar 2;17:1775236. doi: 10.3389/fimmu.2026.1775236 (PMC12989616; doi:10.3389/fimmu.2026.1775236)

A

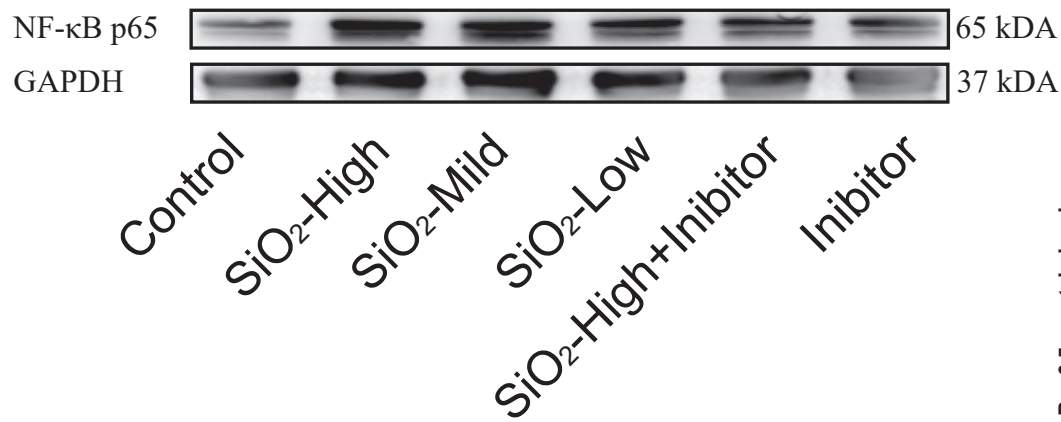

B

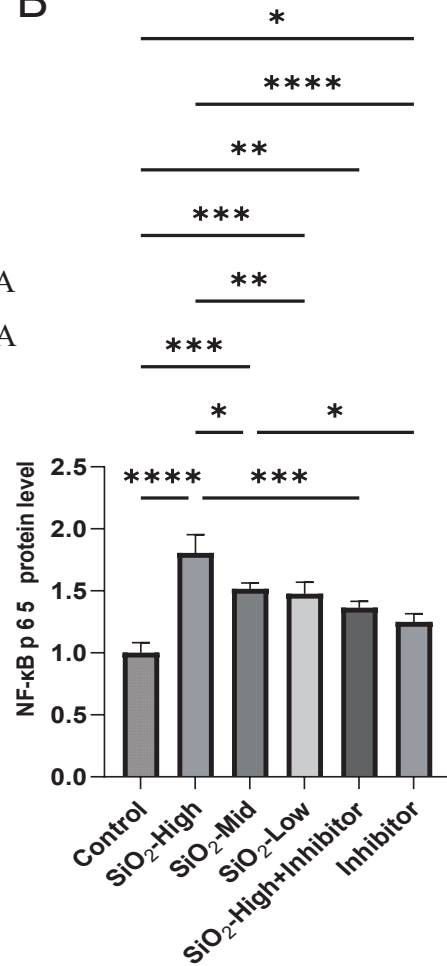

Supplement: Supplementary Figure 1 — NF-κB activation verification. [file Image1.pdf]
